# MD simulation of high-resolution X-ray structures reveals post-translational modification dependent conformational changes in HSF-DNA interaction

**DOI:** 10.1007/s13238-016-0331-0

**Published:** 2016-11-23

**Authors:** Han Feng, Sheng Wang, Ling Guo, Avinash S. Punekar, Rudolf Ladenstein, Da-Cheng Wang, Wei Liu

**Affiliations:** 1 National Laboratory of Biomacromolecules, Institute of Biophysics, Chinese Academy of Sciences, Beijing, 100101 China; 2 Key Laboratory of Molecular Biophysics of the Ministry of Education, College of Life Science and Technology, Huazhong University of Science and Technology, Wuhan, 430074 China; 3 Institute of Immunology, The Third Military Medical University, Chongqing, 400038 China; 4 Department of Biosciences and Nutrition, Karolinska Institutet NOVUM, 14183 Huddinge, Sweden

**Dear Editor,**

Heat shock factors (HSFs) constitute a transcription factor family playing regulatory roles in maintaining cellular protein homeostasis or mediating cell differentiation and development (Akerfelt et al., 2010, Bjork and Sistonen 2010, Westerheide et al., 2012). Some human diseases such as cancer and neurodegeneration are often linked with malfunction of HSFs (Dai et al., 2007, Neef et al., 2011, Scherz-Shouval et al., 2014). The human HSF family consists four members: HSF1-4, which exhibit tissue-specific expression profiles and possess unique but overlapping functions. HSF1 is the major regulator of the heat shock response, while HSF2 is more associated with development and cell differentiation. These two HSFs display similar module organization in their amino acid sequences. From the N- to C-terminus, there are a DNA-binding domain (DBD), an oligomerization domain, a regulatory domain and a transactivation domain. DBD, which is responsible for recognizing and binding target genes, is the most conserved and the only structurally characterized domain to date (Littlefield and Nelson 1999, Jaeger et al., 2016, Neudegger et al., 2016).

Both HSF1 and HSF2 undergo extensive post-translational modifications (PTMs) such as phosphorylation, acetylation and SUMOylation, in an activation-attenuation cycle. Two PTMs occurring in the DBD of HSFs, i.e. acetylation of K80 in HSF1 and SUMOylation of K82 in HSF2, have been demonstrated to play crucial roles in the regulatory mechanism of HSFs. The acetylation is believed to promote the release of HSF1 from the target gene in the attenuation phase (Westerheide et al., 2009), while the biochemical consequence of the SUMO modification is still a matter of debate as either strengthening or weakening the HSF2-DNA interaction was observed in different studies (Xing et al., 2005, Anckar et al., 2006). Despite the significance of these PTMs, the precise dynamic process and detailed structural effects inducted by the modifications remain unclear. In this study, we used combined techniques of X-ray crystallography and molecular dynamics (MD) simulations to address these questions.

We firstly purified and crystallized the the DBDs from both human HSF1 and HSF2 as previously described (Feng et al., 2016), and also co-crystallized HSF1-DBD in complex with a 12-bp ds-DNA with the tail-to-tail orientation. These crystal structures were determined and refined at atomic resolutions (1.32–1.70 Å). The wing motif (residues 83-98 in HSF1 or 75–90 in HSF2) that is highly unstructured in most DBDs crystallized so far was fortunately well resolved in one monomer of either HSF1 or HSF2 in our structures. Comparison with the previously reported structures revealed that our structures adopt basically identical fold but display significant differences in the wing motif, which could account for the difference of PTM patterns between HSF1 and HSF2 (Anckar et al., 2006, Jaeger et al., 2016). Detailed structural descriptions and comparisons are given in the Supplemental results.

Acetylation of K80 in HSF1 and SUMOylation of K82 in HSF2 are PTMs occurring in the wing motif of DBD, both having been demonstrated to play important regulatory roles in HSF biology (Akerfelt et al., 2010, Bjork and Sistonen 2010). These modifications were expected to create significant structural effects to the HSF-DNA interactions (Xing et al., 2005, Westerheide et al., 2009). High-resolution DBD structures comprising the intact wing motif determined in our study served as good starting models for studying the structural effects of these important modifications by molecular dynamics (MD) simulation.

By contrast to the reference system containing an unmodified DBD, the ds-DNA bound by acetylated DBD dramatically tilted during simulation (Fig. 1), though its conformation remained rather steady (Fig. S5). Synchronized with the DNA tilt, acetylated K80 was moving away from the bound DNA, which was resulted from entropy-driven repulsion between the neutralized lysine and the negative phosphate backbone. As a consequence, strand β3 twisted synchronizing with the DNA tilt, both of which reached to the maximum extent at 23.8 ns (Fig. 1 and Movie S1). Meanwhile, the flexible wing loop became evidently more contracted than the unmodified DBD.

**Figure 1:**
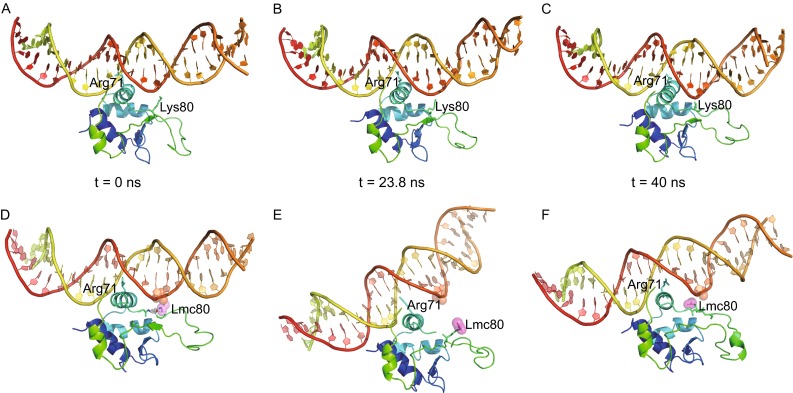
**MD simulation of an acetylated DBD in HSF1 bound to DNA containing a GAA repeat**. (A–C) Temporary states of the reference system comprising unmodified DBD retrieved at 0 (A), 23.8 (B) and 40 ns (C). (D–F) Temporary states of the experimental system comprising modified DBD retrieved at 0 (D), 23.8 (E) and 40 ns (F). The side chain of acetylated K80, labelled as Lmc80, is highlighted by pink spheres. The phosphate group in DNA backbone that is supposed to interact with K80 is highlighted by an orange sphere.

After that time point, the simulation process entered into the second phase. The opened space resulting from DNA inclination allowed acetylated K80 to approach to the helix-turn-helix motif again. Its neutralized side chain, however, was buried in a hydrophobic network surrounded by M66, V70 and F99 until the end of simulation. Formation of this local hydrophobic core induced a significant conformational change at the distant tip of the wing where a short 3_10_ helix was formed accordingly. The bound DNA duplex slightly tilted back, but could not restore to the optimum binding position (Fig. 1 and Movie S1).

In the whole simulation process, the protein-DNA interactions considerably decreased compared with the reference system. R79, for example, which hydrogen-bonded to the phosphate backbone in the context of non-modification, dissociated from DNA after 16 ns (Fig. S5). The major protein-DNA contacts occurring at the major groove were also significantly weakened because of DNA inclination. Interestingly, R71, the key amino acids responsible for site-specific interactions, remained in close contact with the G of GAA throughout the simulation, but its guanidine group flipped, allowing swapping of NH1 and NH2 at ca. 11 ns after the DNA molecule had started to tilt. We thus suppose that the two macromolecules likely dissociate each other in the period from 11 to 16 ns, when major protein-DNA interactions have vanished or diminished.

Overall considering, acetylation of K80 in HSF1 brought about a dramatic tilt of the bound DNA duplex and a flip of the guanidine moiety of R71, both of which strongly diminished HSF1-DNA interactions (Figs. 1 and S5). Although the system could re-equilibrate after the rearrangement of modified K80 and neighbouring residues, we believe that HSF1 and DNA dissociate in reality, which accounts for the promoted release of HSF1 from the target genes in the attenuation phase of HSR (Westerheide et al., 2009).

The simulation of SUMOylated HSF2 bound to ds-DNA could also be divided into two distinct phases. During the first 11 ns, the SUMO2 moiety displayed marked conformational flexibility with respect to DBD and DNA (Fig. S6), and in addition, it strongly tended to depart from DBD, which was likely driven by the necessity of systemic entropy reduction (Fig. 2 and Movie S2). Such spatial departure of SUMO2 and DBD agrees well with a previous NMR study showing the absence of a non-covalent interface between them (Tateishi et al., 2009). In the second phase, the system became equilibrated, with the SUMO2 moiety, in particular, showing much less structural flexibility and just slightly moving closer to DBD (Fig. 2).

**Figure 2:**
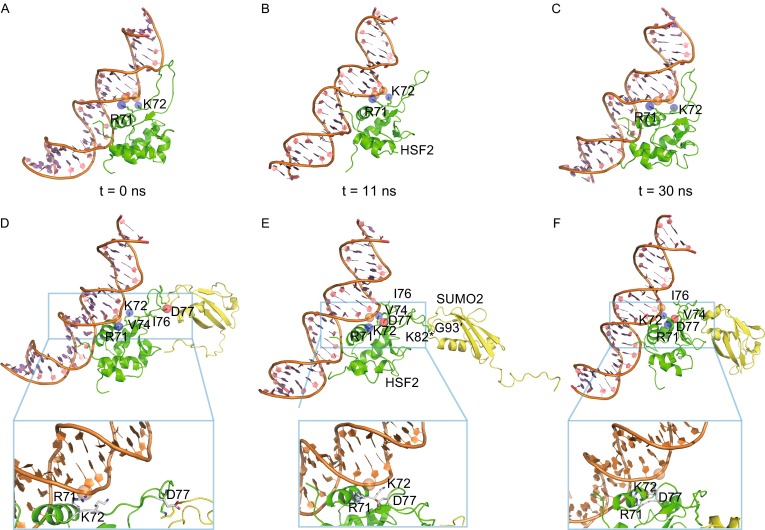
**MD simulation of a SUMOylated DBD in HSF2 bound to DNA containing a GAA repeat**. (A–C) Temporary states of the reference system comprising unmodified DBD retrieved at 0 (A), 11 (B) and 30 ns (C). (D–F) Temporary states of the experimental system comprising modified DBD retrieved at 0 (D), 11 (E) and 30 ns (F). The side chains of R71 and K72 are highlighted by purple spheres; the side chain of D77 is highlighted by a red sphere; the phosphate group in DNA backbone that is supposed to interact with R71 is highlighted by an orange sphere. Closer views of (D–F) are given below the corresponding panels.

In the complete simulation process, high flexibility of the SUMO2 moiety produced profound structural effect to the attached DBD, especially to the wing motif. Residues close to the N-terminus of this loop in an unmodified DBD such as V74, I76 and D77 adopted rather extended conformations even after long-time equilibration. In contrast, positional shifts and interior conformational changes of SUMO2 forced this region to contract and rearrange to form a helical conformation (Fig. 2 and Movie S2). A significant consequence of this rearrangement was the approach of D77 to R71, a well-conserved residue engaged to protein-DNA interactions through electrostatic contacts or hydrogen bonds in the reference system (Figs. 2 and S6). A tight hydrogen bond was then formed between the side chains of R71 and D77 and remained until the end of the simulation, which effectively dragged the positively charged R71 side chain away from the DNA backbone. As a result of this strong charge-charge interaction, the original contact between R71 and DNA was completely abolished, although a neighbouring lysine residue (K72) remained in contact with DNA throughout the time frame of the simulation (Fig. S6).

In addition, the DNA molecule underwent two subtle but significant conformational changes during simulation. By comparison with the initial structure, it rotated along the double helical axis by ca. 15°, and more strikingly, widening of the major groove occurred (Fig. S6). These changes, however, did not much affect the protein-DNA contacts occurring in the major groove including base-specific interactions between GAA and R63, as the side chain of R63 was always placed at hydrogen bonding distance with the guanine base. On the other side, minor groove narrowing happened simultaneously with major groove widening. Although a narrower minor groove often enhances local electrostatic potential of DNA towards a more negative potential and favours the insertion of an arginine side chain (Rohs et al., 2009), the only Arg residue in the vicinity (R71), however, was tethered by D77 and thus unable to contact the minor groove of DNA. Based on the simulated process, we suppose that the conjugated SUMO moiety more likely introduces unfavourable effects on HSF2-DNA interactions through the conformational changes in the wing loop and the abolishment of the interaction of residue R71 with DNA.

In summary, high flexibility of SUMO2 conjugated to HSF2 was observed in our simulation, consistent with an earlier NMR experiment (Tateishi et al., 2009). Nonetheless, different from their observation, our simulation also revealed significant changes in the DBD with the wing motif in particular. Apparently, the entropy driven movement of SUMO2 poised a strong driving force on the wing of HSF2-DBD, which led to refolding of this motif and the formation of a tight hydrogen bond between D77 and R71 (Fig. 2). This divergence, however, may not be contradictory as NMR experiment and MD simulation are performed in incomparable time scales. The NMR study revealed an overall effect of SUMOylation in a time regime from minutes to hours while our simulation uncovered dynamical changes occurring in nanoseconds. From the simulation, we also observed a structural effect to DNA conferred by SUMOylation as well, including rotation along the duplex axis and the major groove widening. Based on these observed dramatic changes, we assume that SUMOylation of K82 in HSF2 more likely negatively modulates the interaction with DNA albeit more weakly than acetylation of K80 in HSF1. Although our conclusion agrees with that drawn from the NMR study (Tateishi et al., 2009), the inhibitory mechanism postulated from both studies seems inconsistent. Tateishi *et al*. speculated that the SUMO attachment inhibits HSF2-DNA interaction through a randomly distributed steric interference, but the simulation performed in our study clearly indicates that the conformational changes of DBD caused by the SUMO modification are more likely responsible for the inhibitory effect.

## Electronic supplementary material

Below is the link to the electronic supplementary material.

Supplementary material 1 (MPG 12257 kb)

Supplementary material 2 (MPG 10378 kb)

Supplementary material 3 (PDF 2007 kb)
