# Supplementary material for: MD simulation of high-resolution X-ray structures reveals post-translational modification dependent conformational changes in HSF-DNA interaction
Source: Protein Cell. 2016 Nov 23;7(12):916–20. doi: 10.1007/s13238-016-0331-0 (PMC5205663; doi:10.1007/s13238-016-0331-0)
Supplement: Supplementary file 3 — Supplementary material 3 (PDF 2007 kb) [file 13238_2016_331_MOESM3_ESM.pdf]

Supplemental materials

MD simulation of crystal structures reveals post-translational modification dependent conformational changes in heat shock factor - DNA interaction

Han Feng[1§], Sheng Wang[2§], Ling Guo[3], Avinash S. Punekar[4], Rudolf Ladenstein[4], Da-Cheng Wang[1,5] and Wei Liu[4]

[1] Institute of Biophysics, Chinese Academy of Sciences, Beijing, 100101, China
[2] College of Life Science and Technology, Huazhong University of Science and Technology, Wuhan, Hubei, 430074, China
[3] Institute of Immunology, The Third Military Medical University, Chongqing, 400038, China
[4] Department of Biosciences and Nutrition, Karolinska Institutet NOVUM, Huddinge, 14183, Sweden
[5] Foshan University, Guangdong, 528000, China

Table of Contents

Experimental Procedures……………………………………………………2
Protein expression and purification…………………………………………2
Crystallization and diffraction data collection………………………………3
Structure determination and refinement……………………………………4
MD simulation of acetylated HSF1-DBD interacting with DNA………………5
MD simulation of SUMOylated HSF2-DBD interacting with DNA …………6
Supplemental Results……………………………………………………………8
Overall crystal structures…………………………………………………...8
Comparison with the DBD of *K. lactis* HSF…………………………………9
The wing motif………………………………………………………………10
Unspecific HSF1-DNA interactions…………………………………………12
Table S1. X-ray data collection and refinement statistics……………………..14
Figure S1. Overall structures of the DBD in HSF1 and HSF2…...…………..…15
Figure S2. Comparison with the DBD in *K. lactis* HSF...……………………16
Figure S3. The wing motif of HSF1 and HSF2…………………………………17
Figure S4. Unspecific DNA-binding of HSF1…………………………………..18
Figure S5. MD simulation of acetylated HSF1 in complex with DNA…………19
Figure S6. MD simulation of SUMOylated HSF2 in complex with DNA……20
References…………………………………………………………………………21

EXPERIMENTAL PROCEDURES

Protein expression and purification

The nucleotide sequences encoding the DNA-binding domain of human HSF1-DBD (residues 15-120) and HSF2 (residues 7-112) were amplified by PCR using the full-length *hsf1* and *hsf2* genes as the templates, respectively. To improve the solubility of the expressed products, both sequences were inserted into plasmid pMAL-c4E (New England Biolabs) for production of maltose-binding protein (MBP) fusion proteins in *Escherichia coli*. A Tobacco Etch Virus (TEV) protease cleavage site and a His_6-tag were introduced to the N-termini of the destination proteins by PCR amplification. The recombinant plasmids were firstly transformed into *E. coli* strain Rosetta2, and pilot experiments showed that both DBDs were expressed in soluble form even after the removal of the MBP-tag by *in vitro* cleavage. To simplify the purification, an *in vivo* cleavage strategy was used by transforming plasmid pRK603, which contained a TEV protease encoding gene (Kapust and Waugh 2000), into the same strain prior to preparation of competent cells using the classical calcium chloride method. After transformation of the expression plasmids pMAL-HSF1DBD and pMAL-HSF2DBD into this strain, the bacteria were grown in LB medium containing 100 μg/ml ampicillin, 50 μg/ml kanamycin and 34 μg/ml chloramphenicol at 37°C. Expression of the MBP-fusion protein was induced with 0.3 mM isopropyl β-D-1-thiogalactopyranoside (IPTG) for 4 h at 30°C immediately followed by expression of the TEV protease induced by 100 ng/ml anhydrotetracyclin hydrochloride (aTet) for another 2 h at 30°C. The MBP-tag was cut off *in vivo* from the fusion protein along with the intracellular production of the TEV protease.

The same purification protocol was applied for both proteins. Bacterial cells were harvested by centrifugation at 5000 g for 30 min at 4°C, resuspended in the lysis buffer containing 50 mM NaH_2PO_4/Na_2HPO_4, pH 8.0, 500 mM NaCl and 25 mM imidazole, and lysed using a high-pressure crusher at 4°C. After

removal of insoluble debris by centrifugation at 20000 g for 30 min at 4°C, the supernatant was immediately loaded onto a Ni^{2+}-NTA chromatography column (Novagen). The destination protein was eluted with 200 mM imidazole and 5% glycerol contained in the same buffer. The pooled fraction was subjected to buffer exchange with several concentration-dilution cycles before loading onto a HiTrap SP HP 5 ml column (GE Healthcare), which is preequilibrated with 20 mM HEPES, pH 7.5 and 100 mM NaCl. A linear-gradient elution with increasing NaCl concentration from 100 mM to 1.0 M was then applied. Size-exclusion chromatography using a Superdex 75 16/600 column (GE Healthcare) was used to further improve the purity of the protein, with an elution buffer containing 20 mM Tris-HCl, pH 8.0, 150 mM NaCl, 1 mM DTT, 0.2 mM EDTA and 5% glycerol. Both purified proteins were concentrated to 40 mg/ml and stored at -80°C until being used for crystallization.

Crystallization and diffraction data collection

The protein samples of HSF1-DBD and HSF2-DBD were diluted to concentrations of 20 and 15 mg/ml respectively prior to crystallization trials. All crystallization experiments were carried out using the hanging-drop vapour diffusion method at room temperature. The drop in each well was formed by mixing 1 μl protein solution with 1 μl reservoir solution and was equilibrated against 500 μl reservoir solution. Crystals of HSF1-DBD were obtained at 0.2 M Sodium formate, pH 7.2 and 22% w/v PEG 3350, while crystals of HSF2-DBD were grown in 0.1 M Tris, pH 8.4 and 1.25 M potassium sodium tartrate. For crystallization of HSF1-DBD and DNA complex, the protein with a concentration of 40 mg/ml was premixed with a 12 bp DNA in sequence of 5'-GG<u>TTC</u>TA<u>GAA</u>CC-3' (Underlined are the GAA triplets in HSE repeats) immediately before the crystallization trials with a molar ratio of protein:DNA = 1:1.5. The cocrystals were grown in 6% ethylene glycol, 0.1 M sodium citrate, pH 3.8 and 12% w/v PEG6000.

The HSF1-DBD crystals used for data collection were dipped into cryoprotectant (reservoir solution with increased concentration of PEG 3350 to 25% w/v) for 15 s before flash-cooling in a stream of liquid nitrogen, while the HSF2-DBD crystals and HSF1-DNA cocrystals were directly mounted in nylon cryoloops (Hampton Research) and flash-cooled in the same way. X-ray diffraction data collection for HSF1-DBD, HSF1-DNA HSF2-DBD was performed on beamline BL17U1, BL18U1 and BL19U1 respectively, at Shanghai Synchrotron Radiation Facility (SSRF) in China. The collected data for HSF1-DBD were evaluated using *HKL2000* (Otwinowski and Minor 1997), while the data for HSF1-DNA and HSF2-DBD were processed using *XDS* (Kabsch 2010). Further details of HSF2-DBD purification, crystallization and data collection were given in another report (Feng et al., 2016).

Structure determination and refinement

The crystal structure of HSF1-DBD was determined by means of molecular replacement using the *Kluyveromyces lactis* HSF-DBD structure (chain B in the PDB entry of 3HTS) (Littlefield and Nelson 1999) as a search model. Automatic structure determination using *Phaser* (Bunkoczi et al., 2013) failed to give a clear solution until the search model was modified with side chain truncation from the Cβ atoms using *Chainsaw* (Stein 2008) in the *CCP4* program suite (Winn et al., 2011). After automatic model building using *Phenix.autobuild* (Terwilliger et al., 2008), the structure was refined using *phenix.refine* (Afonine et al., 2012) with several rounds of manual remodeling between refinement cycles using the modeling toolkit *Coot* (Emsley et al., 2010). The structure of HSF2-DBD was solved by molecular replacement as well using the refined HSF1-DBD structure as a search model, and refined in the same way. The same protein model and a standard B-form DNA duplex generated by using *Coot* were used as search models for molecular replacement of the HSF1-DNA structure. All structures were validated using *MolProbity* (Chen et al., 2010). Statistics from the data collection and structure

refinement are summarized in Table 1. All figures representing the refined structures were prepared using the molecular visualization program *Pymol* (Schrodinger 2010).

Modeling and MD simulations of acetylated HSF1-DBD interacting with ds-DNA

A 25-bp B-form ds-DNA fragment comprising a single GAA repeat in the middle part was generated using *Nucgen* from the *Amber14* package (Case et al., 2015). A model mimicking the protein-DNA complex was obtained by aligning the refined HSF1-DBD structure to chain B in the PDB entry of 3HTS (Littlefield and Nelson 1999), and replacing chain A (the crystallized DNA in complex with *Kluyveromyces lactis* HSF-DBD in 3HTS) with the modeled 25-bp ds-DNA by superimposing the GAA repeats in both. Acetylated K80 in HSF1-DBD was modeled by replacing one hydrogen atom of ε-NH2 with CH_3CO- group. The force field of this non-standard lysine residue (hereafter named as LMC) was derived from calculation of the restrained electrostatic potential (RESP) partial atomic charges using the *GAMESS* program package (Schmit et al., 1993) with HF/6-31G(d) as the basis set. The output values obtained from the GAMESS were used as an input for the antechamber to derive RESP partial atomic charges for the LMC residue. These suitable parameters of LMC were used for later simulation including all GAFF atom types and RESP partial atomic charges of LMC residue.

Molecular dynamics (MD) simulations were performed in parallel using the Amber14 package (Case et al., 2015) for the ensembles containing either wild type or acetylated HSF1-DBD using a five-step protocol including ensemble construction, minimization, heating, equilibration and production. The program suite Amber 14 was used to perform the simulations, with force field parameters from ff14SB and ff99bsc0 for protein and DNA, respectively. The two ensembles were put into truncated octahedrons with explicit solvent fully

filled. The TIP3 water model was used for explicit simulation, with 24498 and 21264 water molecules filled in the boxes containing acetylated and unmodified HSF1 respectively. To neutralize the charges of two ensembles, 48 and 47 Na^+ ions were added into these water boxes, respectively.

After the ensemble building, three runs of simulation including a single run of the unmodified system and two independent runs from the same starting condition but different random seeds were carried out. All simulation calculations were parallel performed on NIVIDA Graphics card including GTX 690 and GTX 780. At first, two 10 ps minimization simulations were conducted with whole solutes and DNA strands fixed to clean the clashes between solutes. Then the ensembles were experienced by 200 ps heating simulation processes until the temperature up to 300 K. This time the whole solutes were fixed with stronger force constants to avoid explosion of systems. After the heating processes, ensembles were equilibrated during a 1 ns simulation. Finally, 40 or 50 million steps of NPT simulation were performed in production with step size of 1 fs. Subsequently, another run of simulation for the acetylated system from the same starting model but with a different seed and longer simulation time (50 ns) was performed in order to test the reproducibility of the trajectories. Trajectory analysis was done by using the imbedded tools in *Amber* 14. The final conformations were extracted from long production simulations and aligned with each other to analyze the interaction differences and conformation changes.

Modeling and MD simulations of SUMOylated HSF2-DBD interacting with ds-DNA

A similar modeling protocol was used in building a model where HSF2-DBD bound to a 25-bp DNA comprising a single GAA repeat in the middle part. A SUMO2 model was obtained by removing the 2 C-terminal residues (V94 and Y95) from a reported NMR structure (PDB entry 2AWT). To mimic

SUMOylation of K82 in HSF2, the SUMO2 model was then docked to the HSF2-DNA model with constraints of positioning its C-terminus located within covalent bonding distance to the ε-NH2 of K82 in HSF2 using *ZDOCK* (Chen et al., 2003). The resultant model thus contained an isopeptide bond connecting K82 in HSF2 and G93 in SUMO2, which were hereafter referred to as K82* and G93*, respectively. The force field parameters for these two isopeptide-bridging amino acids were extracted from the docking model with methylation at α-NH2 and α-COOH of this "bipeptide" to mimic the electric environment in protein context. The GAMESS package (Schmit et al., 1993) was used to calculate the electrostatic potential (ESP), with HF/6-31G(d) as the basis set, and the output values served as an input for the antechamber to derive RESP partial atomic charges for K82*.

Similar to the HSF1 simulations, the simulations of these systems were performed in parallel using the Amber14 package (Case et al., 2015) for the ensembles containing either wild type or SUMOylated HSF2-DBD using a five-step protocol including ensemble construction, minimization, heating, equilibration and production. The two ensembles were put into truncated octahedrons with explicit solvent fully filled. The TIP3 water model was used for explicit simulation, with 31648 and 21648 waters in the solvating boxes containing SUMOylated and unmodified HSF2 respectively. To neutralize the charges of two ensembles, 50 and 47 Na^+ ions were added into these boxes, respectively.

Similar to the HSF1 systems, three runs of simulation including a single run of the unmodified system and two independent runs from the same starting condition but different random seeds were carried out after the ensemble building. The simulation calculations were performed by following a same protocol as that for acetylated HSF1 bound to ds-DNA, except for 30 or 50 million steps in the production.

SUPPLEMENTAL RESULTS

Overall crystal structures

The structures presented here belonged to different space groups and were refined with good model quality, as indicated by the crystallographic and stereochemical parameters given in Table 1. The asymmetric unit of the HSF1-DBD crystal contained only one protein monomer (Fig. S1a), while those in the cocrystal with DNA and the HSF2-DBD crystal both comprised four protein monomers (Fig. S1b, c). In the structures except HSF1-DBD, dimers of dimer were formed with the two monomers in each dimer related by two-fold non-crystallographic symmetry, but displaying different dimeric interfaces. In tetramer arrangement, however, there was no symmetry relating the two dimers in both structures. Obviously, crystal packing rather than molecular properties and shapes were most likely responsible for such diversity of oligomer organization of HSF-DBDs observed in these crystals.

In all these structures, most amino acids even including some histidine residues within the N-terminal His-tag are present in the refined models. The highly flexible wing loop (residues 83-98 in HSF1 or 75-90 in HSF2) invisible in almost all reported structures of HSF-DBD (Harrison et al., 1994, Littlefield and Nelson 1999, Jaeger et al., 2016, Neudegger et al., 2016) was also poorly defined in the electron density of most protein monomers and had to be omitted from the models. Even so, an intact wing loop in monomer C was observed in the complex structure of HSF1-DBD bound to DNA (Fig. S1b), and similarly in monomer D in the HSF2-DBD structure (Fig. S1c). It seems likely that the conformation of these loops is well fixed by contacts from neighboring protein monomers. Hereafter, we use these two monomers to represent the DBD structures of human HSF1 and HSF2, respectively.

The DBD copies in both the HSF1 structures displayed little deviations (RMSD = 0.213 ~ 0.488 Å among them), and so did the HSF2-DBD structure (RMSD =

0.190 ~ 0.360 Å). Furthermore, the DBD structures between the two human HSFs showed closely resembled fold to each other (RMSD = 0.266 ~ 0.417 Å), and to the recent published structures (PDB ID 5D5U and 5D8K) (Jaeger et al., 2016, Neudegger et al., 2016) as well (RMSD = 0.569 ~ 0.699 Å). All these comparisons indicated insignificant conformational changes of HSF-DBDs whether bound to DNA or not. The DBD scaffold exhibits a canonical winged helix-turn-helix motif centred at the structural core, with two additional helices (α1 and α4) serving as N- and C-terminal extensions (Fig. S1d, e).

It is noteworthy that the structure of HSF2-DBD was determined at 1.32 Å, the resolution of which made alternative rotamer conformations became visible in electron density. The side chains of a total of 27 amino acids were defined in more than one rotamer, which otherwise led to significant residual $F_{obs} - F_{calc}$ density and unreasonable R_{free} values. The hydroxyl oxygen of S35 in monomer A, for example, could be undoubtedly interpreted by dual conformations (Fig. S1f).

Comparison with the DBD of *K. lactis* HSF

Among the functional modules, the DNA binding domain is the most conserved domain in HSF. The DBDs of human HSF1 and HSF2 share 49% and 50% sequence identity with *K. lactis* HSF, respectively (Fig. S2a), though with much lower overall homology for the full-length sequence. Structure comparison also revealed very similar architecture among these transcription factors. Superimposition of HSF1-DBD or HSF2-DBD onto a previously reported structure of *K. lactis* HSF (PDB ID 3HTS) (Littlefield and Nelson 1999) resulted in good structure overlay with RMSDs of 0.933 or 0.881 Å, respectively (Fig. S2b, c). In *K. lactis* HSF, a proline residue (P237) is unusually present at an internal position in helix α2, leading to the formation of a helical kink. This proline seems insignificant for protein function or stability but critically required for folding kinetics (Hardy and Nelson 2000). Notably, the proline residue is

conserved in human HSFs (P58 in HSF1 and P50 in HSF2) (Fig. S2a), in which a similar kinked helix is formed (Fig. S2b, c), suggesting a common role of this amino acid.

Compared to the partial DBD structure in PDB entry 3HTS, a complete DBD architecture including the wing motif and the intact C-terminal conformation was observed in our and the recently published structures (Jaeger et al., 2016, Neudegger et al., 2016). Strikingly, a two-turn 3_{10} helix (α4) occurs in the DBD of both human HSFs (residues 109-114 in HSF1 and 101-106 in HSF2). The presence of such a helix is quite unusual as a typical 3_{10} helix contains only a single turn in protein structures, which would otherwise be unstable. In DBD structures, however, the C-terminal helix is well stabilized by the interactions from other structural elements, in particular the N-terminal helix (α1). The hydrophobic residues present in these two helices, e.g. L19 and W23 in α1 and L112 in α4 in HSF1, show close contacts (Fig. S2d). Furthermore, two phenylalanine residues preceding the C-terminal helix (e.g. F99 and F104 in HSF1) are involved in a hydrophobic cluster with the other two phenylalanines located in the helix-turn-helix motif (F44 and F78) (Fig. S2e). All these hydrophobic interactions surrounding helix α4 renders the C-terminal conformation rather rigid, which may constrain the conformational freedom of DBDs in an HSF trimer.

The wing motif

Unlike most DNA-binding proteins containing a winged helix-turn-helix motif, the wing in *K. lactis* HSF did not interact with the bound DNA duplex (Littlefield and Nelson 1999), which was very recent observed in recently reported structures as well (Jaeger et al., 2016, Neudegger et al., 2016). This motif, however, was proposed as an important structural element for full activity of *K. lactis* HSF (Cicero et al., 2001), the HSE-binding specificity of human HSF1 and the responses to heat stress (Ahn et al., 2001). The wing is highly

unstructured in most DBDs crystallized so far, but fortunately it was well resolved in one monomer of either HSF1 or HSF2 in our structures (Fig. S3a, b).

Intriguingly, the wing topology presented here was not identical to that observed in the recent published structures (Fig. S3c, d). In those structures (PDB ID 5D5V and 5D8K) where the wing puckered upward with a topology partially constrained by a hydrogen bond formed between the side chains of two conserved amino acids (H83 and E98 in HSF1 or H75 and E90 in HSF2). By contrast, the wing revealed in our structures adopted a more open conformation probably because of the lack of this hydrogen bond. Although the location of the glutamic acid was conserved among these structures, the histidine residue was positioned 6 Å away, far from hydrogen bonding distance. Notably, two positively charged residues closely upstream the conserved histidine (R79 and K80 in HSF1 or R71 and K72 in HSF2) contacted the DNA phosphate backbone in the published structures. We hence speculate that DNA-binding may be the major driving force for the formation of the hydrogen bond at the stem part of the wing, which renders this loop less flexible.

SUMOylation of K82 in the wing of HSF2 has been proven as an important PTM in regulating the activity of this transcription factor (Goodson et al., 2001, Xing et al., 2005). Although a lysine residue (K91) is present at the corresponding position in HSF1, it seems impossibly to be modified by SUMO (Anckar et al., 2006, Tateishi et al., 2009, Jaeger et al., 2016). A previous study has suggested that the residues downstream K82, in particular G87 and P88, were required for SUMOylation in HSF2-DBD (Anckar et al., 2006). These two amino acids are replaced by an aspartic acid (D96) in the HSF1 sequence (Fig. S2a), which takes the same spatial position as P88 in HSF2 (Fig. S3e). The presence of this negatively charged residue seems to well constrain the flexibility of K91 by forming a salt bridge between them, and as a result, K91 in

HSF1 adopts a partially buried conformation. In contrast, K82 in HSF2 is significantly more solvent exposed, and hence more accessible to a SUMO ligase in the cell. (Fig. S3e). Thus, the structural deviations revealed in these two DBDs could well explain the difference of SUMO modification between the two transcription factors.

Unspecific HSF1-DNA interactions

In this study, we cocrystallized the DBD of HSF1 with two tail-to-tail orientated HSE repeats, which was identical to that used in the structure of PDB 3HTS (Littlefield and Nelson 1999) or 5D5U (Neudegger et al., 2016). Unfortunately, we failed to observe a sequence-specific binding architecture in this high-resolution structure. Despite the protein-DNA stoichiometry remaining 1:1 in that crystal, only two DBD copies appeared bound to DNA (Fig. S1b). Instead of being inserted into the major groove of DNA, the recognition helix of either monomer A or C lay along the double helix albeit over the major groove (Fig. S4a). With such an orientation, conserved residues in the recognition helix and the preceding short loop, such as H63, R71 and Y76, failed to contact any nucleobases through direct or water-bridging hydrogen bonds, but instead interact with the phosphate backbone only.

A similar case has been observed in the cocrystal of *K. lactis* HSF-DBD bound to a 12-mer DNA, in which however the recognition helix also failed to contact DNA (Littlefield and Nelson 2001). Apparently, site-specific DNA binding was most likely blocked by crystal packing interaction in both cases. Different from those structures, however, major protein-DNA contacts were mediated by the recognition helix in our structure, which allows us to speculate that this binding manner might not be completely physiologically irrelevant. For most DNA-binding proteins, the first step in DNA recognition and binding would be the formation of unspecific contacts dominantly driven by electrostatic interactions (Ohlendorf and Matthew 1985). In this sense, a possible

representation of such a binding architecture might be an initial phase in the dynamic process of HSF-HSE recognition and binding, and starting from this given state, the relative protein-DNA orientation is subsequently fine-tuned until final specific binding architecture is reached.

Recent ChIP-seq experiments have demonstrated genome-wide presence of highly diverse HSEs with variable sequences, length and orientation of the nGAAn repeats (Guertin and Lis 2010, Gonsalves et al., 2011). The gap- or step-type HSEs identified in the yeast genome (Sakurai and Takemori 2007, Sakurai and Enoki 2010), for example, both represent non-canonical HSEs where contiguous nGAAn repeats are disrupted by insertion of a non-consensus pentanucleotide unit (Fig. S4b, c). In these cases, one DBD in a single trimer of HSF is supposed to bind DNA in an unspecific manner, although the other two DBDs must form sequence-specific interactions with the GAA triplets. As another scenario, the DNA binding architecture revealed in our structure might represent such a non-site-specific binding mode between one DBD in an HSF trimer and a non-consensus HSE repeat (Fig. S4b, c).

Table S1. Data collection and refinement statistics

	HSF1	HSF1-DNA	HSF2
**Data collection**			
PDB ID	5HDG	5HDN	5HDK
Space group	*I222*	$P2_1$	$P2_12_12_1$
Cell			
a, *b*, *c* (Å)	48.61, 64.42, 68.77	39.42, 127.37, 55.27	65.66, 67.26, 93.25
α, β, γ (°)	90, 90, 90	90, 100.55, 90	90, 90, 90
Resolution (Å)	32.21-1.7 (1.73-1.70)	41.35-1.68 (1.72-1.68)	38.32-1.32 (1.39-1.32)
R_{sym}	0.12 (0.527)	0.047 (0.477)	0.066 (0.657)
$\langle I/\sigma(I)\rangle$	15.1 (4.1)	15.9 (2.2)	18.4 (3.6)
Completeness (%)	99.5 (99.8)	98.4 (88.6)	99.8 (99.8)
Redundancy	7.1 (7.3)	3.64 (3.45)	12.0 (11.2)
Wilson plot B	14.5	30.8	15.2
**Refinement**			
No. reflections	12180	60860	97250
R_{work} / R_{free}	0.191 / 0.218	0.174 / 0.202	0.153 / 0.174
No. atoms			
Protein	789	3332	3497
DNA		972	
Ions	1 (Na^+)	21 (CIT, Na^+)	18 (K^+, Na^+, Cl^-)
Water	149	710	543
B-factors			
Protein	30.10	30.52	24.18
DNA		34.53	
Ions	22.83	27.79	19.27
Water	42.96	42.97	37.97
R.m.s. deviations			
Bond lengths	0.003	0.006	0.016
Bond angles (°)	0.753	0.943	1.539
Ramachandran			
Favored (%)	95.6	95.9	98.2
Allowed (%)	4.4	4.1	1.8
Outliers (%)	0	0	0

*Values in parentheses are for highest-resolution shell.

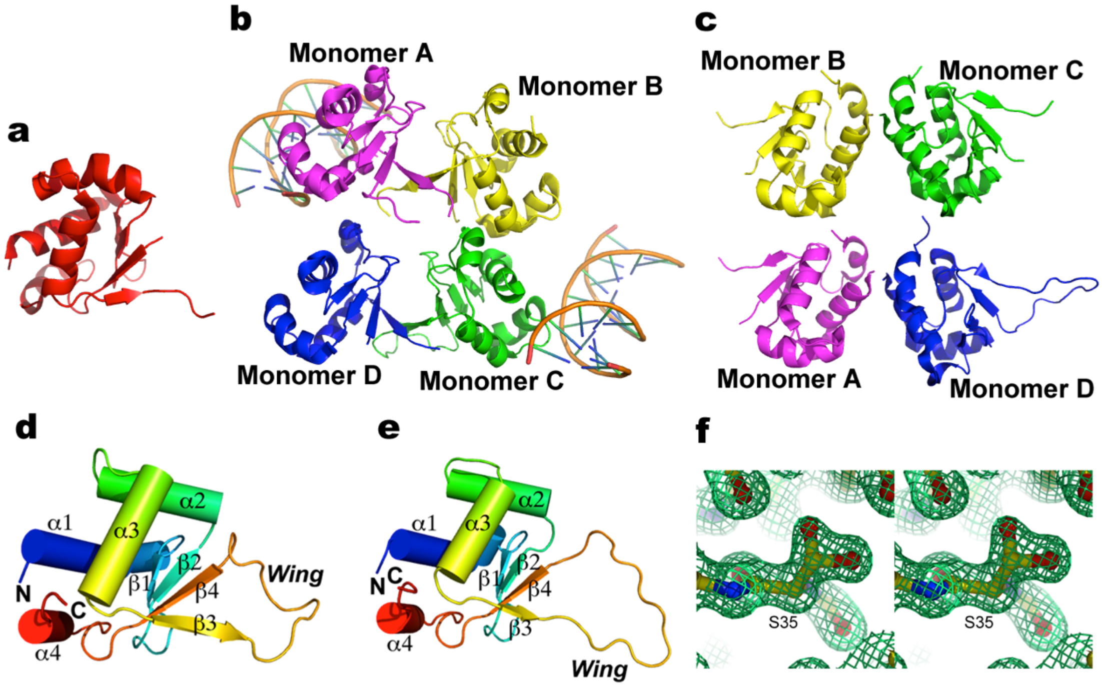

Figure S1. Overall structures and subunit architecture of the DNA-binding domain in HSF1 and HSF2. Protein monomers in the asymmetric unit of HSF1-DBD (**a**), HSF1-DBD in complex with DNA (**b**) and HSF2-DBD (**c**) are represented by ribbon models. The DBD topology of HSF1 (**d**) and HSF2 (**e**) is colored in rainbow manner from the N- (blue) to the C-terminus (red). (**f**), An example of alternative side chain rotamer defined in the $2f_o - f_c$ density (contour = 1.0) of HSF2-DBD.

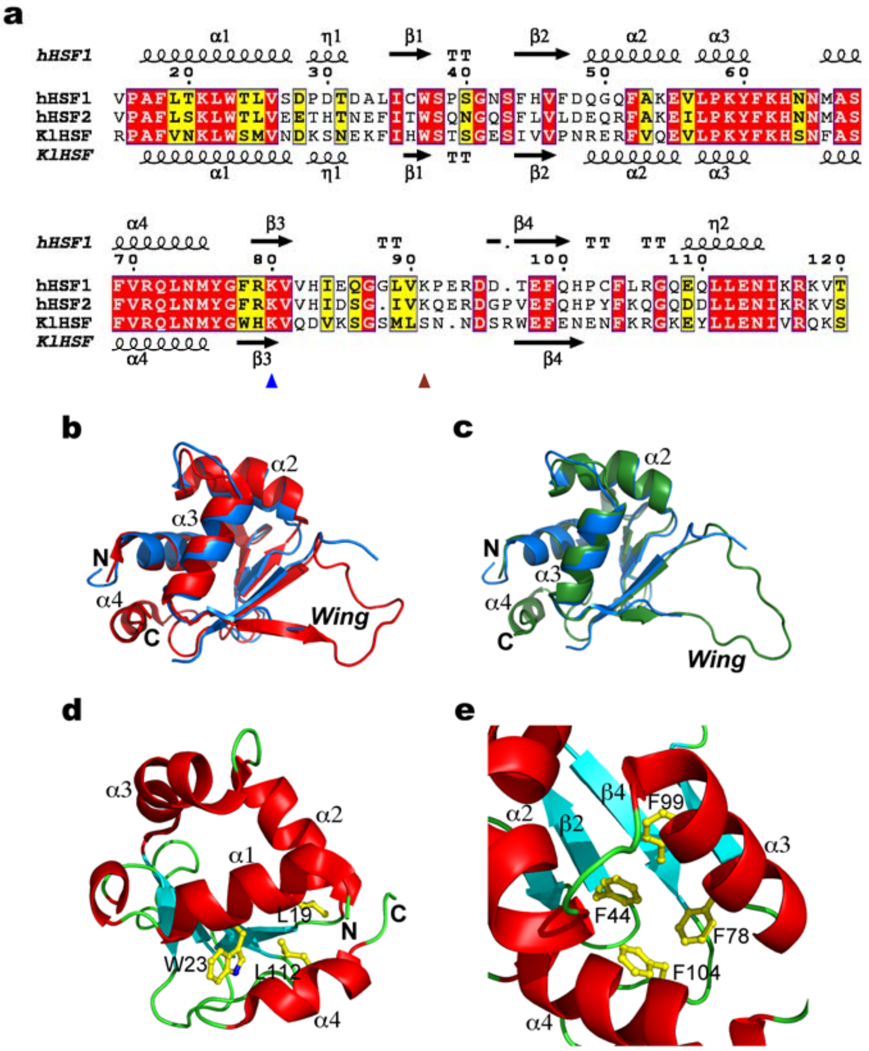

Figure S2. Comparison with the DNA-binding domain in *K. lactis* HSF (PDB ID 3HTS) (Littlefield and Nelson 1999). (**a**), Sequence alignment of the DBD among human HSF1, HSF2 and *K. lactis* HSF. (**b**) and (**c**), Structural superimposition of *K. lactis* HSF onto human HSF1 (**b**) or HSF2 (**c**). (**d**) and (**e**), the C-terminal conformation of HSF1 is stabilized by hydrophobic interactions between helices α1 and α4 (**d**), and a phenylalanine cluster with the involvement of the loop preceding helix α4 and the helix-turn-helix motif (**e**). The hydrophobic residues shown in stick models are all conversed in HSF2.

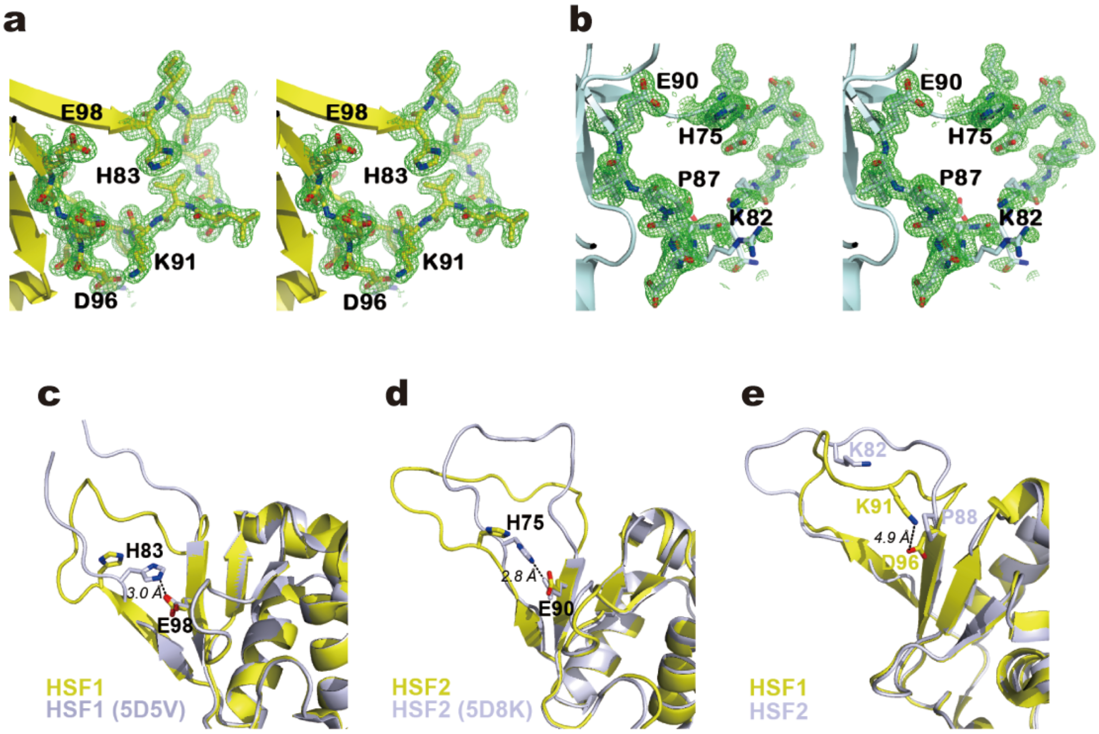

Figure S3. The wing motif of HSF1 and HSF2. (**a** and **b**), Stereo view of the wing in HSF1 (**a**) and HSF2 (**b**) fitting in $2F_{obs} - F_{calc}$ density contoured at 1.0 σ. The wing motif is represented in stick model and the omit density maps are displayed as green mesh (**c**-**e**), Comparison of the wing in HSF1 (**c**) and HSF2 (**d**) determined in our structure with recent published structures (Jaeger et al., 2016, Neudegger et al., 2016), and (**e**), Comparison between HSF1 and HSF2. Hydrogen bonds in (**c**) and (**d**) and the salt bridge in (**e**) are represented by dash lines.

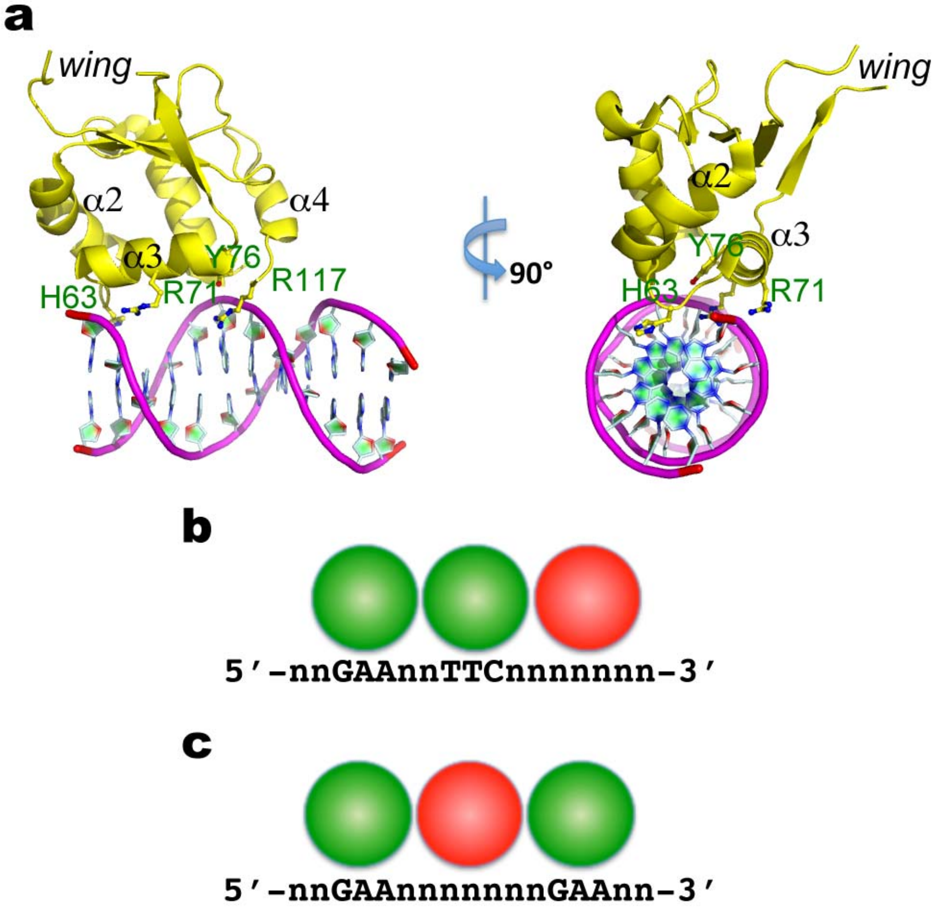

Figure S4. Unspecific DNA-binding of HSF1. (**a**), The HSF1-DNA interactions revealed in the cocrystal structure of HSF1-DBD bound to a tail-to-tail oriented HSE. (**b**) and (**c**), Potential unspecific binding of one DBD in an HSF trimer to HSEs containing non-consensus pentanulcleotide repeats such as the gap- (**b**) or step-type HSE (**c**) (Sakurai and Takemori 2007, Sakurai and Enoki 2010). Site-specific and unspecific bound DBDs are denoted by green and red circles, respectively.

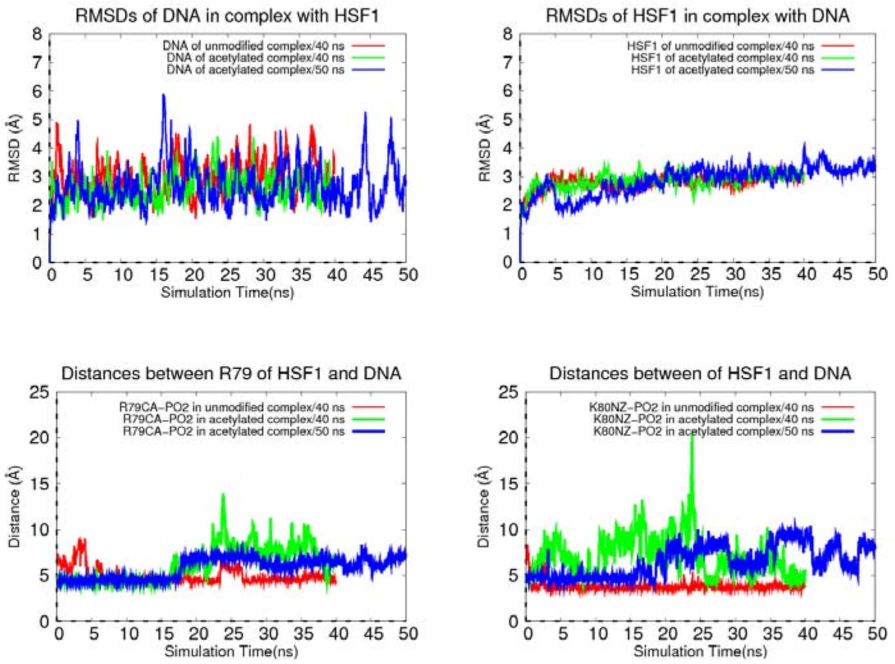

Figure S5. RMSD and distance distribution of MD simulations of K80-acetylated HSF1 in complex with DNA.

a

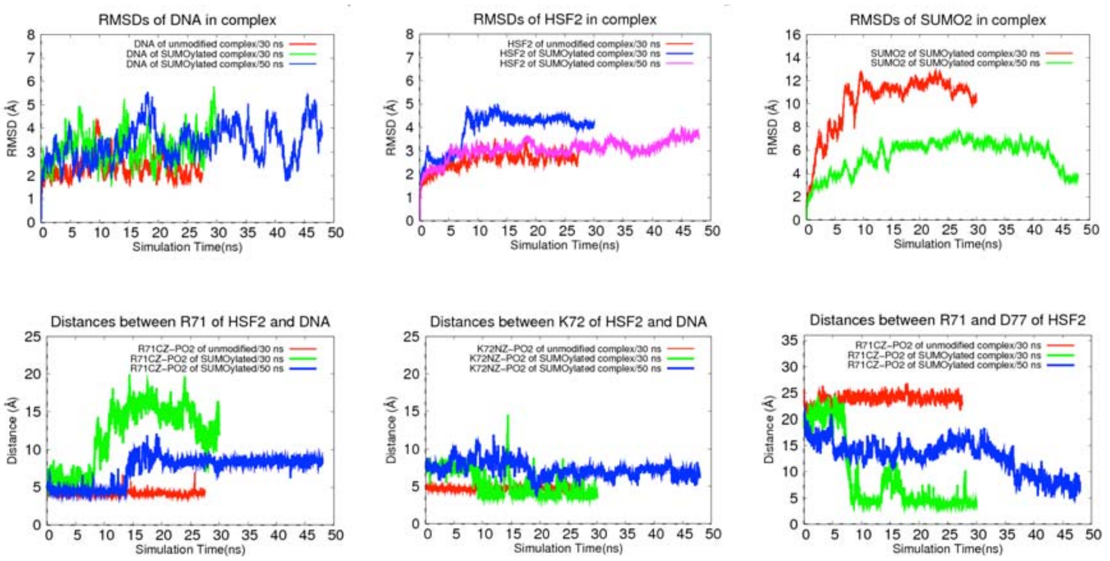

b

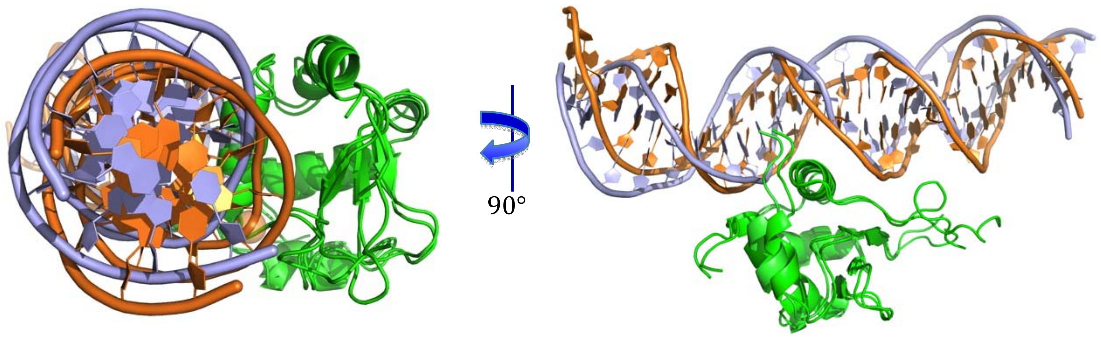

Figure S6. MD simulations of K82-SUMOylated HSF2 in complex with DNA. (**a**), RMSD and distance distribution in the simulation processes. (**b**), The conformational change of DNA highlighted by superimposition of the initial (brown) and the final (lightblue) structures of the simulation. The superimposed DBDs are denoted in green ribbon model, while the SUMO-2 moiety was hided for clarity.

REFERENCES

Afonine, P. V., R. W. Grosse-Kunstleve, N. Echols, J. J. Headd, N. W. Moriarty, M. Mustyakimov, T. C. Terwilliger, A. Urzhumtsev, P. H. Zwart, P. D. Adams (2012). "Towards automated crystallographic structure refinement with phenix.refine." Acta crystallographica. Section D, Biological crystallography **68**(Pt 4): 352-367.

Ahn, S. G., P. C. Liu, K. Klyachko, R. I. Morimoto, D. J. Thiele (2001). "The loop domain of heat shock transcription factor 1 dictates DNA-binding specificity and responses to heat stress." Genes Dev **15**(16): 2134-2145.

Anckar, J., V. Hietakangas, K. Denessiouk, D. J. Thiele, M. S. Johnson, L. Sistonen (2006). "Inhibition of DNA binding by differential sumoylation of heat shock factors." Mol Cell Biol **26**(3): 955-964.

Bunkoczi, G., N. Echols, A. J. McCoy, R. D. Oeffner, P. D. Adams, R. J. Read (2013). "Phaser.MRage: automated molecular replacement." Acta Crystallogr D Biol Crystallogr **69**(Pt 11): 2276-2286.

Case, D. A., J. T. Berryman, R. M. Betz, D. S. Cerutti, I. Cheatham, T.E., T. A. Darden, R. E. Duke, T. J. Giese, H. Gohlke, A. W. Goetz, N. Homeyer, S. Izadi, P. Janowski, J. Kaus, A. Kovalenko, T. S. Lee, S. LeGrand, P. Li, T. Luchko, R. Luo, B. Madej, K. M. Merz, G. Monard, P. Needham, H. Nguyen, H. T. Nguyen, I. Omelyan, A. Onufriev, D. R. Roe, A. Roitberg, R. Salomon-Ferrer, C. L. Simmerling, W. Smith, J. Swails, R. C. Walker, J. Wang, R. M. Wolf, X. Wu, D. M. York, P. A. Kollman (2015). AMBER 2015. San Francisco, University of California.

Chen, R., L. Li, Z. Weng (2003). "ZDOCK: an initial-stage protein-docking algorithm." Proteins **52**(1): 80-87.

Chen, V. B., W. B. Arendall, 3rd, J. J. Headd, D. A. Keedy, R. M. Immormino, G. J. Kapral, L. W. Murray, J. S. Richardson, D. C. Richardson (2010). "MolProbity: all-atom structure validation for macromolecular crystallography." Acta Crystallogr D Biol Crystallogr **66**(Pt 1): 12-21.

Cicero, M. P., S. T. Hubl, C. J. Harrison, O. Littlefield, J. A. Hardy, H. C. Nelson (2001). "The wing in yeast heat shock transcription factor (HSF) DNA-binding domain is required for full activity." Nucleic Acids Res **29**(8): 1715-1723.

Emsley, P., B. Lohkamp, W. G. Scott, K. Cowtan (2010). "Features and development of Coot." Acta Crystallogr D Biol Crystallogr **66**(Pt 4): 486-501.

Feng, H., W. Liu, C. Wang da (2016). "Purification, crystallization and X-ray diffraction analysis of the DNA-binding domain of human heat-shock factor 2." Acta Crystallogr F Struct Biol Commun **72**(Pt 4): 294-299.

Gonsalves, S. E., A. M. Moses, Z. Razak, F. Robert, J. T. Westwood (2011). "Whole-genome analysis reveals that active heat shock factor binding sites are mostly associated with non-heat shock genes in Drosophila melanogaster." PLoS One **6**(1): e15934.

Goodson, M. L., Y. Hong, R. Rogers, M. J. Matunis, O. K. Park-Sarge, K. D. Sarge (2001). "Sumo-1 modification regulates the DNA binding activity of heat

shock transcription factor 2, a promyelocytic leukemia nuclear body associated transcription factor." J Biol Chem **276**(21): 18513-18518.
Guertin, M. J., J. T. Lis (2010). "Chromatin landscape dictates HSF binding to target DNA elements." PLoS Genet **6**(9): e1001114.
Hardy, J. A., H. C. Nelson (2000). "Proline in alpha-helical kink is required for folding kinetics but not for kinked structure, function, or stability of heat shock transcription factor." Protein Sci **9**(11): 2128-2141.
Harrison, C. J., A. A. Bohm, H. C. Nelson (1994). "Crystal structure of the DNA binding domain of the heat shock transcription factor." Science **263**(5144): 224-227.
Jaeger, A. M., C. W. t. Pemble, L. Sistonen, D. J. Thiele (2016). "Structures of HSF2 reveal mechanisms for differential regulation of human heat-shock factors." Nat Struct Mol Biol **23**(2): 147-154.
Kabsch, W. (2010). "Xds." Acta Crystallogr D Biol Crystallogr **66**(Pt 2): 125-132.
Kapust, R. B., D. S. Waugh (2000). "Controlled intracellular processing of fusion proteins by TEV protease." Protein Expr Purif **19**(2): 312-318.
Littlefield, O., H. C. Nelson (1999). "A new use for the 'wing' of the 'winged' helix-turn-helix motif in the HSF-DNA cocrystal." Nat Struct Biol **6**(5): 464-470.
Littlefield, O., H. C. Nelson (2001). "Crystal packing interaction that blocks crystallization of a site-specific DNA binding protein-DNA complex." Proteins **45**(3): 219-228.
Neudegger, T., J. Verghese, M. Hayer-Hartl, F. U. Hartl, A. Bracher (2016). "Structure of human heat-shock transcription factor 1 in complex with DNA." Nat Struct Mol Biol **23**(2): 140-146.
Ohlendorf, D. H., J. B. Matthew (1985). "Electrostatics and flexibility in protein-DNA interactions." Adv Biophys **20**: 137-151.
Otwinowski, Z., W. Minor (1997). "Processing of X-ray diffraction data collected in oscillation mode." Method Enzymol **276**: 307-326.
Sakurai, H., Y. Enoki (2010). "Novel aspects of heat shock factors: DNA recognition, chromatin modulation and gene expression." FEBS J **277**(20): 4140-4149.
Sakurai, H., Y. Takemori (2007). "Interaction between heat shock transcription factors (HSFs) and divergent binding sequences: binding specificities of yeast HSFs and human HSF1." J Biol Chem **282**(18): 13334-13341.
Schmit, M. W., K. K. Baldridge, J. A. Boatz, S. T. Elbert, S. Koseki, N. Matsunaga, K. A. Nguyen, S. Su, T. L. Windus, M. Dupuis, J. A. Montgomery Jr (1993). "General Atomic and Molecular Electronic Structure System." J Comput Chem **14**(11): 1347-1363.
Schrodinger, LLC (2010). The PyMOL Molecular Graphics System, Version 1.3r1.
Stein, N. (2008). "CHAINSAW: a program for mutating pdb files used as templates in molecular replacement." J Appl Cryst **41**(3): 641-643.
Tateishi, Y., M. Ariyoshi, R. Igarashi, H. Hara, K. Mizuguchi, A. Seto, A. Nakai, T. Kokubo, H. Tochio, M. Shirakawa (2009). "Molecular basis for SUMOylation-dependent regulation of DNA binding activity of heat shock

factor 2." J Biol Chem **284**(4): 2435-2447.
Terwilliger, T. C., R. W. Grosse-Kunstleve, P. V. Afonine, N. W. Moriarty, P. H. Zwart, L. W. Hung, R. J. Read, P. D. Adams (2008). "Iterative model building, structure refinement and density modification with the PHENIX AutoBuild wizard." Acta Crystallogr D Biol Crystallogr **64**(Pt 1): 61-69.
Winn, M. D., C. C. Ballard, K. D. Cowtan, E. J. Dodson, P. Emsley, P. R. Evans, R. M. Keegan, E. B. Krissinel, A. G. Leslie, A. McCoy, S. J. McNicholas, G. N. Murshudov, N. S. Pannu, E. A. Potterton, H. R. Powell, R. J. Read, A. Vagin, K. S. Wilson (2011). "Overview of the CCP4 suite and current developments." Acta crystallographica. Section D, Biological crystallography **67**(Pt 4): 235-242.
Xing, H., D. C. Wilkerson, C. N. Mayhew, E. J. Lubert, H. S. Skaggs, M. L. Goodson, Y. Hong, O. K. Park-Sarge, K. D. Sarge (2005). "Mechanism of hsp70i gene bookmarking." Science **307**(5708): 421-423.
